# Gene Expression Analysis of the Endocannabinoid System in Presymptomatic APP/PS1 Mice

**DOI:** 10.3389/fphar.2022.864591

**Published:** 2022-03-18

**Authors:** Laura Vidal-Palencia, Carla Ramon-Duaso, Jose Antonio González-Parra, Arnau Busquets-Garcia

**Affiliations:** “Cell-Type Mechanisms in Normal and Pathological Behavior” Research Group, IMIM-Hospital del Mar Medical Research Institute, PRBB, Barcelona, Spain

**Keywords:** Alzheimer Disease, APP/PS1 mice, brain-region analysis, mouse behaviour, CB1 receptors, sex-dependent

## Abstract

Alzheimer’s disease (AD) is the most common type of dementia and neurodegeneration. The actual cause of AD progression is still unknown and no curative treatment is available. Recently, findings in human samples and animal models pointed to the endocannabinoid system (ECS) as a promising therapeutic approach against AD. However, the specific mechanisms by which cannabinoid drugs induce potential beneficial effects are still undefined. For this reason, it is required a full characterization of the ECS at different time points of AD progression considering important factors such as sex or the analysis of different brain regions to improve future cannabinoid-dependent therapies in AD. Thus, the main aim of the present study is to expand our knowledge of the status of the ECS in a presymptomatic period (3 months of age) using the AD mouse model APP/PS1 mice. First, we evaluated different behavioral domains including anxiety, cognitive functions, and social interactions in male and female APP/PS1 mice at 4 months of age. Although a mild working memory impairment was observed in male APP/PS1 mice, in most of the behaviors assessed we found no differences between genotypes. At 3 months of age, we performed a characterization of the ECS in different brain regions of the APP/PS1 mice considering the sex variable. We assessed the expression of the ECS components by quantitative Real-Time Polymerase Chain Reaction in the hippocampus, prefrontal cortex, hypothalamus, olfactory bulb, and cerebellum. Interestingly, gene expression levels of the type-1 and type-2 cannabinoid receptors and the anabolic and catabolic enzymes, differed depending on the brain region and the sex analyzed. For example, CB1R expression levels decreased in both hippocampus and prefrontal cortex of male APP/PS1 mice but increased in female mice. In contrast, CB2R expression was decreased in females, whereas males tended to have higher levels. Overall, our data indicated that the ECS is already altered in APP/PS1 mice at the presymptomatic stage, suggesting that it could be an early event contributing to the pathophysiology of AD or being a potential predictive biomarker.

## 1 Introduction

Dementia, which is characterized by several behavioral alterations that affect patients’ daily life, is one of the greatest global challenges for health in the 21st century (Livingston et al., 2017; Mullane and Williams, 2020). There are many different types of dementia, but Alzheimer’s disease (AD) is the most common one (Livingston et al., 2017; Jacobs et al., 2018; Ray and Buggia-Prevot, 2021). AD is a progressive, age-dependent process, characterized by specific molecular hallmarks that accompany the behavioral dysfunctions including cognitive and social alterations (Lane et al., 2018; Avila and Perry, 2020; Coles et al., 2020). The prevalence of AD is increasing as life expectancy is improving, yet the very few treatments available are symptomatic and do not modify disease progression (Livingston et al., 2017; Ray and Buggia-Prevot, 2021).

There are several progressive stages of AD based on the evolution of the behavioral and molecular deficits (Braak and Braak, 1991; Guo et al., 2017; Jack et al., 2018; Jacobs et al., 2018; Dibattista et al., 2020). An interesting period in this progression is the presymptomatic stage, which occurs between the earliest pathogenic event of AD and the appearance of behavioral symptoms such as cognitive deficits. This stage could serve to identify early molecular alterations and novel interventions to delay, detect or counteract AD pathophysiology (Maccarrone et al., 2018; Qin et al., 2020). Indeed, animal models have become a useful tool to characterize possible molecular changes prior to any behavioral alteration (Caselli and Reiman, 2012). Thus, an accurate understanding of AD pathogenesis is required by untangling the possible molecular pathways that lead to behavioral disturbances.

Taking into account the repertoire of possible molecular mechanisms involved in AD, the endocannabinoid system (ECS) has emerged as a potential therapeutic approach against this neurodegenerative disease (Cristino et al., 2020). The ECS is a retrograde neuromodulatory system involved in a plethora of physiological processes such as the regulation of brain homeostasis, neurotransmitter release, and synaptic transmission processes. By doing this, the ECS can modulate some behavioral outputs such as learning and memory, motor coordination, energy balance, and olfactory functions (Castillo et al., 2012; Busquets-Garcia et al., 2015; Cristino et al., 2020). Briefly, this system comprises at least two G protein-coupled receptors (GPCRs), known as type-1 and type-2 cannabinoid receptors (CB1R and CB2R), the lipid-derived signaling molecules anandamide (AEA) and 2-arachidonoylglycerol (2-AG), and the endocannabinoid metabolizing enzymes (Castillo et al., 2012; Busquets-Garcia et al., 2015; Roger, 2015; Cristino et al., 2020).

There are still many unresolved questions aiming at better understanding the association between the ECS and AD. Alterations in the expression and/or activity of the different components of the ECS have been described both in AD animal models and in human patients (Aso and Ferrer, 2014; Berry et al., 2020; Cristino et al., 2020). Interestingly, *in vitro* and *in vivo* pharmacological activation of CB1Rs displayed efficacy in reducing the neurotoxic effects of amyloid-β (Aβ) peptide and in counteracting the cognitive impairment found in AD mouse models (Aso et al., 2012; Haghani et al., 2012). Nevertheless, most of these previous results have focused only on particular brain regions (e.g., hippocampus and/or cortex) (Kalifa et al., 2011; Aso et al., 2012; Haghani et al., 2012; Aso et al., 2016; Aso et al., 2018; Medina-Vera et al., 2020) without providing a full characterization of the ECS in other brain regions, and without taking into account potential sex-dependent differences in most of the studies.

As a thorough characterization of the ECS in AD mouse models is required, the main goal of the present study was to analyze the expression of the main components of the ECS in a presymptomatic stage of AD considering the sex differences and brain region-dependent changes using male and female APP/PS1 mice. This AD mouse model recapitulates major features of amyloid and behavioral pathology of AD in a progressive manner (Aso et al., 2016; Jankowsky and Zheng, 2017; Aso et al., 2018). First, we evaluated behavioral and molecular alterations at early stages, confirming that this is a period with mild behavioral impairments and absence of other neuropathological alterations according to previous literature (Bilkei-Gorzo, 2014; Webster et al., 2014; Jankowsky and Zheng, 2017; Kosel et al., 2020; Peng et al., 2021). Second, we characterized the gene expression of the ECS components in APP/PS1 mice at 3 months of age to identify possible sex- and brain region-dependent molecular dysfunctions at early stages of AD pathology.

## 2 Materials and Methods

### 2.1 Animals

Male and female APPswe/PS1ΔE9 (APP/PS1) mice on a C57BL/6J background (#034832-JAX, Jackson Laboratory, Bar Harbor, ME, United States) and their wild-type (WT) littermates were used in this project. This double transgenic animal co-express a chimeric mouse/human amyloid precursor protein (Mo/HuAPP695swe), and the human exon-9-deleted variant of PS1 (PS1-dE9) (Bilkei-Gorzo, 2014; Kosel et al., 2020). To generate APP/PS1 and WT littermates, mice were bred in the Barcelona Biomedical Research Park (PRBB) animal facility (Barcelona, Spain) that has full accreditation from the Association for Assessment and Accreditation of Laboratory Animal Care (AAALAC). Animals were grouped-housed and maintained in a temperature (20–24°C) and humidity (40%–70%) controlled condition under a 12 h light/dark cycle and had *ad libitum* access to food and water. Behavioral studies were performed during the dark cycle by trained researchers that were blind to the different experimental conditions. All the procedures are adhered to the guidelines of the European Directive on the protection of animals used for scientific purposes (2010/62/EU) and approved by the Animal Ethics Committee of the PRBB (CEEA-PRBB, ABG-19-0041) and from the Generalitat de Catalunya (10785).

#### 2.1.1 Mouse Genotyping

Mice were identified by ear snips at P21 (weaning), and the tissue collected was used to determine the genotype by PCR analysis of isolated genomic DNA (gDNA). Briefly, ear samples were digested with proteinase K (Roche Diagnostics, Mannheim, Germany) and gDNA was isolated and amplified using GoTaq^®^ Master Mix (Promega, Madison, WI, United States). Genotyping primers used to detect the APP transgene were obtained from Jackson Laboratories (see Table 1 for sequences). Amplification was performed in 40 cycles, each one consisting of 30 s at 94°C, 1 min at 65°C, and 1 min at 72°C. Genotypes were confirmed by running the samples in a 2.5% agarose gel electrophoresis and visualized with a chemiluminescence system (ChemiDoc XRS+, Bio-Rad). When mice were euthanized at the end of the behavioral experiments, genotypes were re-confirmed using a portion of the tail.

**TABLE 1:** Primers used for mouse genotyping.

Primer	Forward primer (5′-3′)	Reverse primer (5′-3′)
APP transgene	AGG​ACT​GAC​CAC​TCG​ACC​AG	CGG​GGG​TCT​AGT​TCT​GCA​T
Internal Positive Control	CTA​GGC​CAC​AGA​ATT​GAA​AGA​TCT	GTA​GGT​GGA​AAT​TCT​AGC​ATC​ATC​C

### 2.2 Behavioral Evaluation

**FIGURE 1:**
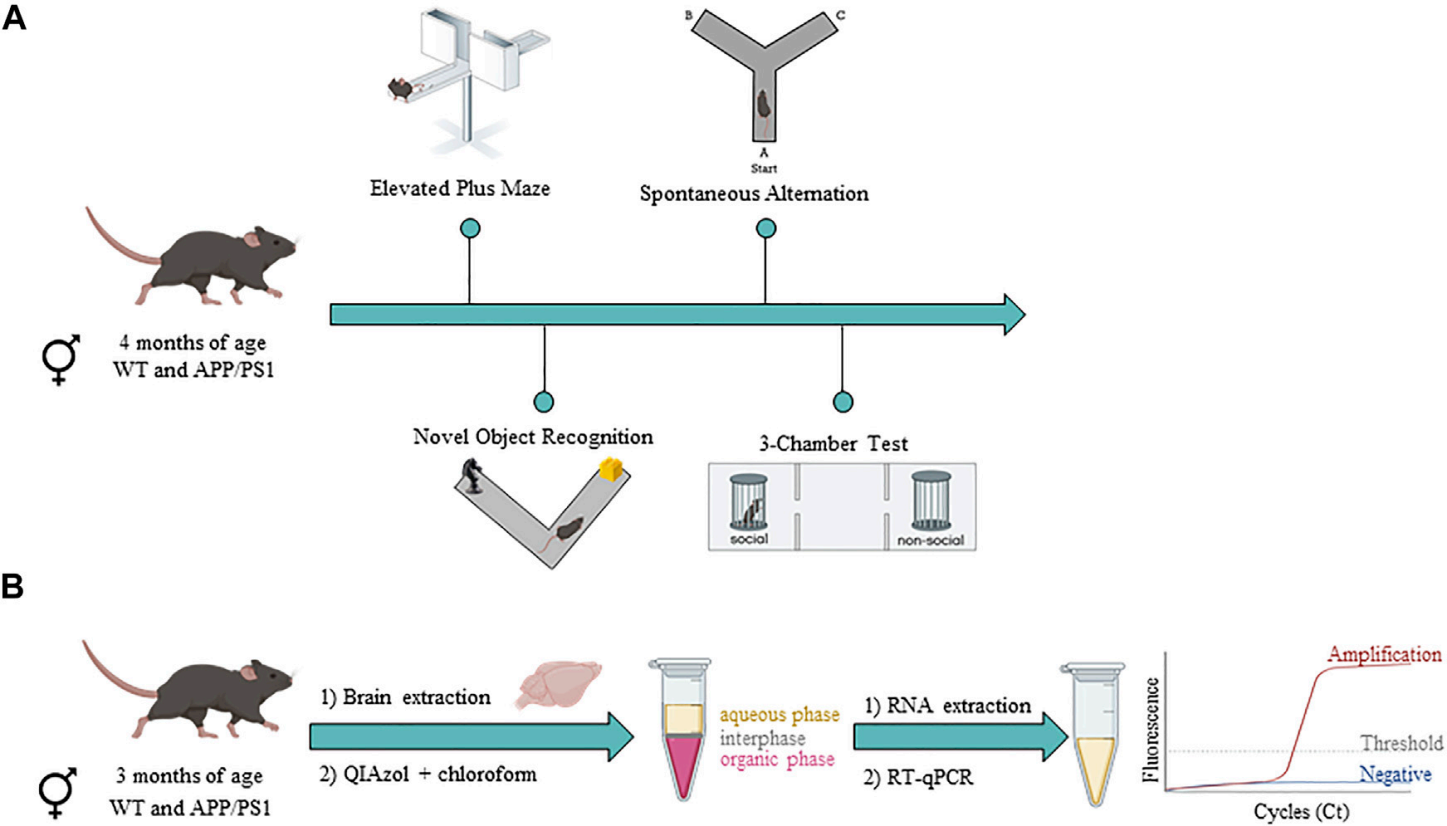
Timeline of experimental procedures in male and female WT and APP/PS1 mouse line. **(A)** Behavioral assessment in WT (male *n* = 9; female *n* = 12) and APP/PS1 (male *n* = 13; female *n* = 11) mice at 4 months of age. **(B)** Gene expression analysis in WT (male *n* = 9–10; female *n* = 9–10) and APP/PS1 (male *n* = 9–10; female *n* = 9–10) mice at 3 months of age. Figure created with BioRender.com.

To assess potential behavioral phenotypes in male and female APP/PS1 mice compared to their WT littermates, we performed a behavioral battery (Figure 1A). Hereby, anxiety-like behavior (Elevated Plus Maze), cognition (Novel Object Recognition test and Spontaneous alternation task), and social behavior (3-chamber test) were assessed at 4 months of age. All experiments were manually analyzed by researchers that were blind to the experimental conditions.

#### 2.2.1 Elevated Plus Maze

Male and female WT and APP/PS1 mice were tested for anxiety-like behavior using the EPM (Walf and Frye, 2007). The EPM consisted of an elevated cross-shaped apparatus with 2 oppositely positioned open arms and 2 enclosed arms delimited by walls 30 cm in height, and a center square (Panlab s.l.u., Barcelona, Spain). The open arms were illuminated by indirect white light at 90 lux while closed arms were set at 15 lux. This behavioral task focuses on the innate response of mice to prefer enclosed and dark spaces and to avoid open and illuminated areas as they perceive it as a possible threatening environment. During the 5-min test, the time spent in open arms were calculated. An entry into an arm was considered when all 4 limbs were positioned within the arm. In general, an anxiety-like state is pointed as an increase in the percentage of time spent in open arms (time open arms*100/300).

#### 2.2.2 Novel Object Recognition Test

Long-term memory was evaluated in male and female WT and APP/PS1 mice using the NOR test in a V-maze apparatus as previously described (Puighermanal et al., 2009; Oliveira da Cruz et al., 2020). This task is based on the fact that animals will explore more time a novel object than a familiar one to satisfy their innate curiosity and exploratory instinct (Webster et al., 2013). The NOR test consisted of three phases of 9 min each separated by 24 h: habituation to an empty maze, familiarization with the presentation of two identical objects, and the test, in which the familiar object is replaced by a novel one. The exploration time of the novel (Tn) and familiar (Tf) objects in the test phase was measured to assess the discrimination index (DI = (Tn − Tf)/(Tn + Tf)) as an outcome of memory recognition. We defined object exploration as when mice oriented themselves towards the object and touch it with the nose.

#### 2.2.3 Y-Maze Spontaneous Alternation Test

Spontaneous alternation activity was calculated as a measure of spatial working memory in male and female WT and APP/PS1 mice. This test was performed in a Y-shaped maze (Panlab s.l.u., Barcelona, Spain) consisting of three arms illuminated at 40 lux and is based on the inherent preference of rodents to explore a new and unvisited arm rather than reexamining a previous investigated one. During the 8 min of the test, the total number of entries and the number of correct alternations were measured as a proxy of spatial working memory. We defined a correct alternation when mice entered into 3 different arms of the maze consecutively and we considered an entry when the 4 paws were within the arm. With these parameters, we calculated the percentage of spontaneous alternation (number of correct alternations/[total number of entries − 2] *100).

#### 2.2.4 Three-Chamber Test

Social behavior can be measured using the three-chamber test. This task was performed in a three-chambered apparatus (Panlab s.l.u., Barcelona, Spain) separated each of the compartments by an open door with an illumination of 70 lux and was divided into two consecutive phases of 10 min each. In the first phase or habituation phase, male and female WT and APP/PS1 mice were allowed to explore the maze with two empty wire cups located at opposite corners of the maze. In the sociability phase, an unfamiliar C57BL/6J mouse (sex- and strain-matched) was placed in one of the wire cups (social cup) while the other remained empty (non-social cup). We measured the interaction time (sniffing) towards the social cup and non-social cup to determine a social index (time sniffing social cup/[time sniffing social cup + time sniffing non-social cup]). An increase in the social index reflects greater sociability.

### 2.3 Molecular Characterization

#### 2.3.1 Quantitative Real-Time PCR Analysis

To characterize the different components of the ECS, a separate group of male and female WT and APP/PS1 mice were sacrificed by cervical dislocation at 3 months of age and different brain regions were manually dissected and stored at −80°C until use (Figure 1B). For each brain region, we applied an extraction method that allowed us to analyze the total RNA fraction. Briefly, brain areas were placed in a Douncer containing 1 ml (for prefrontal cortex, hippocampus, and cerebellum) or 500 µl (for olfactory bulb and hypothalamus) of QIAzol Lysis Reagent. Later, brain homogenates were mixed with 200 µl of chloroform and after centrifugation, we transferred the supernatant (aqueous phase) to a new tube. To isolate total RNA from the aqueous phase, the RNeasy Lipid Tissue Mini Kit (QIAGEN) was used according to the manufacturer’s instructions. The quality and concentration of RNA from each sample was determined by a NanoDrop 1000 Spectrophotometer (Thermo Fisher Scientific) and then all were quickly stored at −80°C until use.

For each brain-region sample, complementary DNA (cDNA) was obtained using the High-Capacity cDNA Reverse Transcription Kit (Applied Biosystems, CA, United States) in a 20-µl reaction tube and stored at −20°C until use. Reverse transcriptase reactions were carried out at 25°C for 10 min, 2 h at 37°C, and followed by 5 min at 85°C. All samples were adjusted with autoclaved Milli-Q water to a final cDNA concentration of 30 ng/μl per sample.

We quantified the gene expression levels of *Cnr1* and *Cnr2*, the enzymes related with the endocannabinoid’s biosynthesis diacylglycerol lipase-alpha (*Dglα*), diacylglycerol lipase-beta (*Dglβ*), and N-acyl phosphatidylethanolamine phospholipase D (*Napepld*), and the ones related with its hydrolysis which are the monoglyceride lipase (*Mgll*) and fatty acid amide hydrolase (*Faah*). Moreover, we analyzed astroglia and microglia activation (or reactivity) via the measurement of the transcriptional levels of Aldehyde Dehydrogenase 1 L1 (*Aldh1l1* gene) and Signal Transducer and Activator of Transcription 3 (*Stat3* gene) for reactive astrocytes, and Allograft Inflammatory Factor 1 (*Aif1* gene) and chemokine (C-X3-C) Receptor 1 (*Cx3cr1* gene) for microglia reactivity. All samples were tested in triplicate for each qRT-PCR, and β-actin was used as an endogenous housekeeping gene to normalize the transcriptional levels of all target genes analyzed. Primers were designed and verified using the primer-BLAST designing tool (see Table 2 for sequences).

**TABLE 2:** Primers used for qRT-PCR experiments.

Primers for ECS characterization			
Name	Gene	Forward primer (5′-3′)	Reverse primer (5′-3′)
β-actin	*Actb*	TCC​ATC​ATG​AAG​TGT​GAC​GT	GAG​CAA​TGA​TCT​TGA​TCT​TCA​T
Signal transducer and activator of transcription 3 (STAT3)	*Stat3*	ACCCAACAGCCGCCGTAG	CAG​ACT​GGT​TGT​TTC​CAT​TCA​GAT
Aldehyde dehydrogenase 1 family (ALDH1L1)	*Aldh1l1*	GCA​GGT​ACT​TCT​GGG​TTG​CT	GGA​AGG​CAC​CCA​AGG​TCA​AA
Allograft inflammatory factor 1 (Aif1)	*Aif1*	GGA​GAC​GTT​CAG​CTA​CTC​TGA​C	CAT​CCA​CCT​CCA​ATC​AGG​GC
CX3C chemokine receptor 1 (CX3CR1)	*Cx3cr1*	CGT​GAG​ACT​GGG​TGA​GTG​AC	GGA​CAT​GGT​GAG​GTC​CTG​AG
CB1 receptor (CB1R)	*Cnr1*	GAA​GCC​TTT​CTT​CAG​CTC​GAC	AAC​AGC​ACA​CTG​GTG​ACT​CC
CB2 receptor (CB2R)	*Cnr2*	TAT​GCT​GGT​TCC​CTG​CAC​TG	GAG​CGA​ATC​TCT​CCA​CTC​CG
Diacylglycerol lipase, alpha (DAGL α)	*Dglα*	AAT​TTG​CGG​ACT​TAC​AAC​CTG​CGG	TCC​CAG​ACA​GGA​AAG​CCA​AGA​TGT
Diacylglycerol lipase, beta (DAGL β)	*Dglβ*	AGG​GAT​GGA​TGT​GAT​CCC​CA	AAC​AGT​AGC​CCG​GGG​AGT​AT
N-acyl phosphatidylethanolamine phospholipase D (NAPE)	*Napepld*	ATG​CAG​AAA​TGT​GGC​TGC​GAG​AAC	ACC​ACC​TTG​GTT​CAT​AAG​CTC​CGA
Monoglyceride lipase (MAGL)	*Mgll*	TGG​CAT​GGT​CCT​GAT​TTC​ACC​TCT	TTC​AGC​AGC​TGT​ATG​CCA​AAG​CAC
Fatty acid amide hydrolase (FAAH)	*Faah*	TAG​CTT​GCC​AGT​ATT​GAC​CTG​GCT	AGG​AAG​TAA​TCG​GGA​GGT​GCC​AAA

qRT-PCR was performed in an Optical 384-well plate with a QuantStudio™ 12K Sequence Detection System (Applied Biosystems, CA, United States) consisting of 2 activation stops (50°C for 2 min, then 95°C for 10 min) followed by 45 cycles of melting (95°C for 15 s) and annealing (60 °C for 1 min). The PCR reaction contained PowerUp SYBR Green Master Mix (Applied Biosystems, CA, United States). The comparative cycle threshold (ΔΔCt) method was applied to determine gene expression relative quantification (RQ) and the results were reported as fold change compared with the control group for each sex.

### 2.4 Statistical Analysis

One-way ANOVA was used to analyze the behavioral assessment at 4 months of age (SPSS Inc., Chicago, IL, United States). For gene expression analysis, data was analyzed considering the genotype differences (male or female APP/PS1 mice compared with its corresponding WT group), and the non-parametric Mann–Whitney test was applied since the data did not follow a normal distribution according to the Shapiro-Wilk test (SPSS Inc., Chicago, IL, United States). In addition, to analyze sex-dependent differences, we calculated the relative difference of fold change in percentage (%) between WT and APP/PS1 mice for males and females and we performed a Mann–Whitney test for statistical analysis. All data are presented as mean ± SEM (Standard Error of the Mean) using the GraphPad Prism 8.0 software (GraphPad Software, La Jolla, CA, United States). Statistical significance was considered at the *p* < 0.05 level.

## 3 Results

### 3.1 Behavioral and Molecular Characterization of APP/PS1 Mice at Early Stages

**FIGURE 2:**
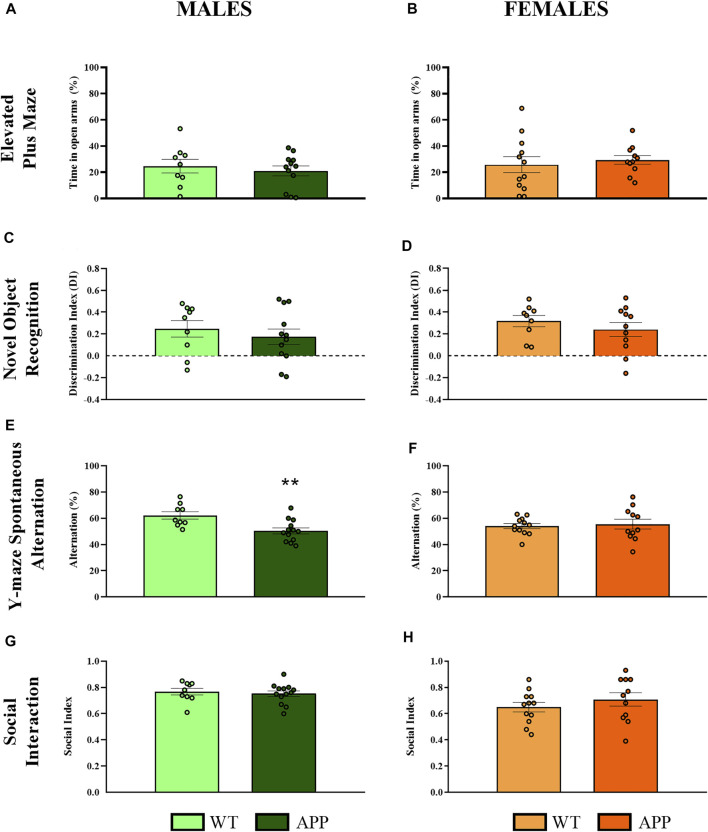
Behavioral evaluation in WT (male *n* = 9; female *n* = 12) and APP/PS1 (male *n* = 13; female *n* = 11) mice at 4 months of age. **(A,B)** Percentage of time spent in open arms to assess anxiety-like behavior in the EPM. **(C,D)** Discrimination index as a long-term memory recognition in the NOR. **(E,F)** Percentage of spontaneous alternation in the Y-maze as a measure of spatial working memory. **(G,H)** Social index as a measure of social interaction using the three-chamber test. Values are the mean ± SEM. **p < 0.01 vs. WT group (one-way ANOVA).

**FIGURE 3:**
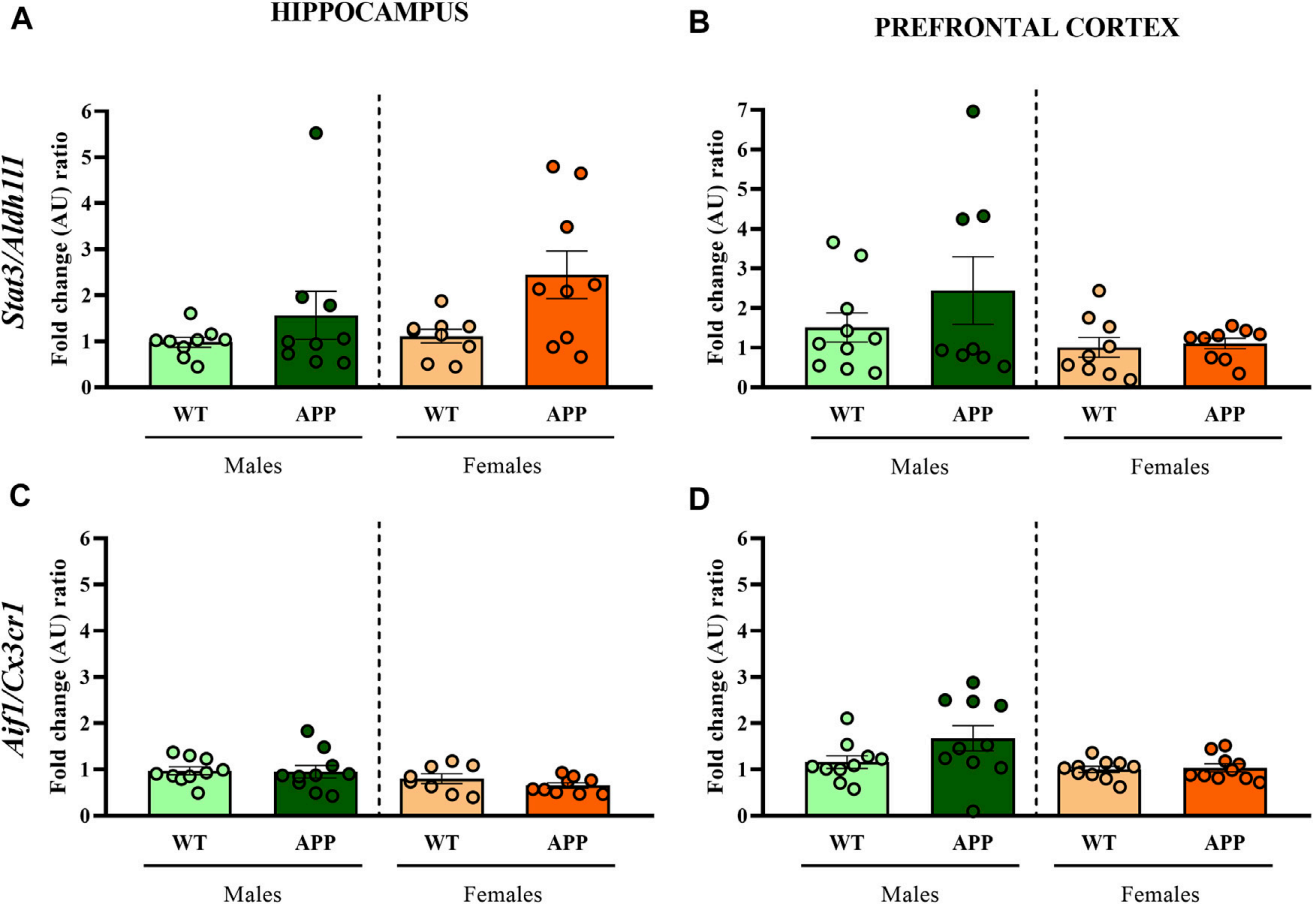
Fold change ratio of *Stat3/Aldh1l1* and *Aif1/Cx3cr1* in WT (male *n* = 10; female *n* = 10) and APP/PS1 (male *n* = 10; female *n* = 10) mice. Fold change ratio of *Stat3/Aldh1l1* (as a proxy for astrogliosis) and *Aif1/Cx3cr1* (as a proxy for microgliosis) in the **(A,C)** hippocampus and **(B,D)** prefrontal cortex respectively, of male and female WT and APP/PS1 mice at 3 months of age. The *X* axis shows the four groups analyzed: male APP/PS1 mice compared with their corresponding WT littermates, and female APP/PS1 mice compared with their control group. The *Y* axis shows the mRNA expression as a fold change ratio. Values are presented as the mean ± SEM; (Mann–Whitney test).

First, we performed a battery of behavioral tests to assess anxiety, cognition and social-related behaviors. In most of the behavioral tasks (i.e., EPM, NOR, or social interaction), no significant differences between genotypes were found (Figure 2). In the spontaneous alternation task, one-way ANOVA revealed a significant effect of genotype between groups [F (1, 19) = 9.8, *p* < 0.005] in males (Figure 2E), but not in females (Figure 2F), suggesting that male APP/PS1 mice displayed significant working memory deficits in comparison with their WT littermates. Overall, the lack of behavioral phenotypes in most of the behavioral tasks and the mild cognitive deficit observed in male APP/PS1 indicates that the stage prior to 4 months of age can be considered as a presymptomatic period in APP/PS1 mice, which might be relevant to study potential molecular alterations that could predict or be involved in AD pathophysiology. Then, we decided to study other molecular alterations that have been associated with AD pathology such as astrogliosis or microgliosis. Comparing the hippocampus and prefrontal cortex of male and female WT and APP/PS1 mice at 3 months of age, any significant difference was observed in the ratio between Stat3/Aldh1l1 (*astrogliosis*) or between Aif1/Cx3cr1 (*microgliosis*) gene expression (Figure 3). These results suggest a lack of these pathological neuroinflammatory processes associated with AD pathology at early stages in APP/PS1 mice.

### 3.2 APP/PS1 Mice Present Alterations in the ECS at 3 Months of Age

To characterize the main components of the ECS at a presymptomatic stage, we analyzed the gene expression of different components of the ECS in WT and APP/PS1 mice at 3 months of age.

#### 3.2.1 Cannabinoid Receptors

**FIGURE 4:**
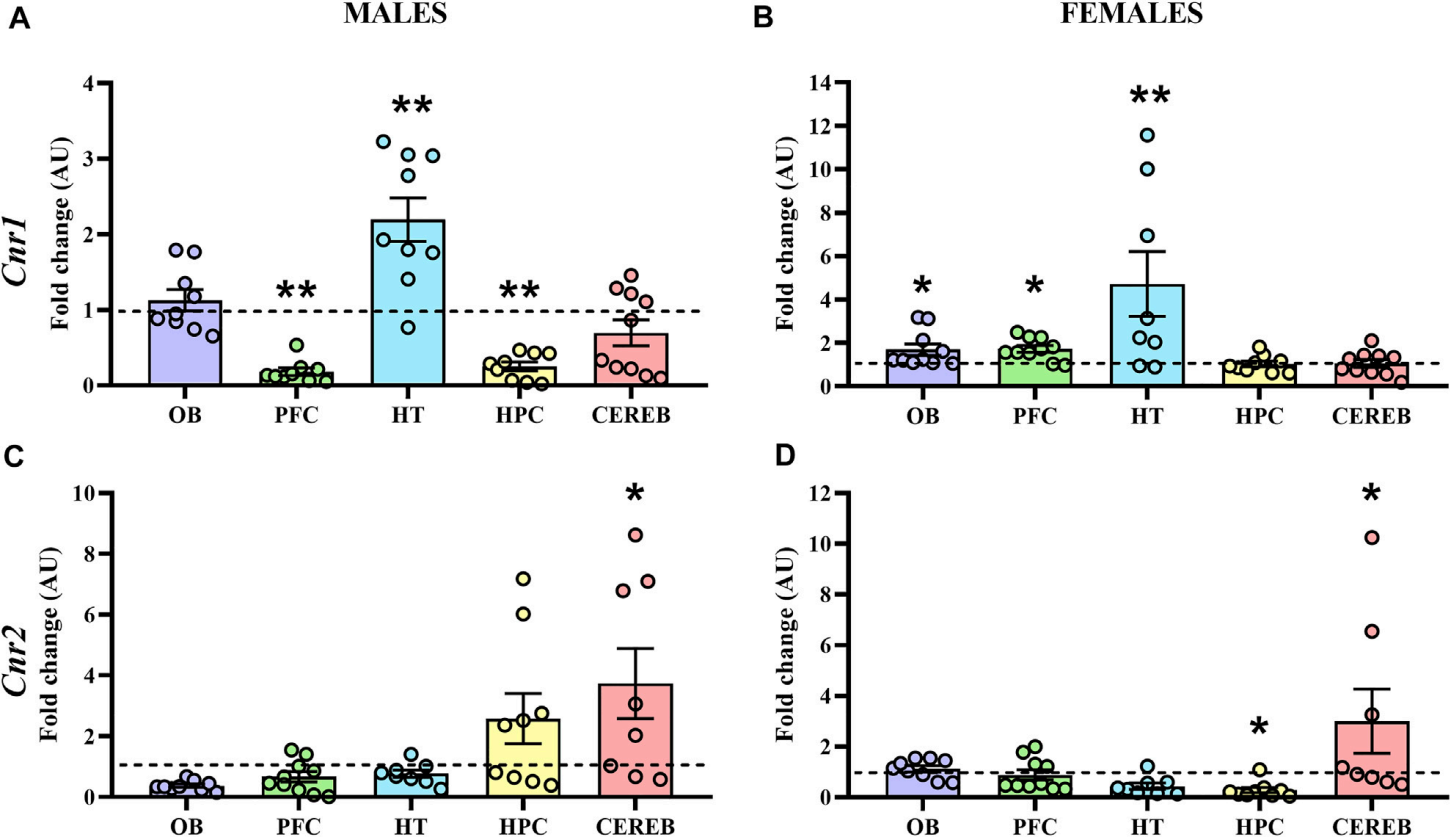
Gene expression of CB1R and CB2R in WT (male *n* = 10; female *n* = 10) and APP/PS1 (male *n* = 10; female *n* = 10) mice at 3 months of age. RT-qPCR assay was performed to analyze the expression of *Cnr1* in **(A)** males and **(B)** females, as well as the expression of *Cnr2* in **(C)** males and **(D)** females. The *X* axis shows the five brain-regions analyzed: olfactory bulb (OB), prefrontal cortex (PFC), hypothalamus (HT), hippocampus (HPC), and cerebellum (CEREB). The *Y* axis shows the mRNA expression as a fold change comparing male and female APP/PS1 mice with its corresponding WT (represented as a dashed line). Values are presented as the mean ± SEM. *p < 0.05, **p < 0.01, vs. WT group (genotype effect); (Mann–Whitney test).

**FIGURE 5:**
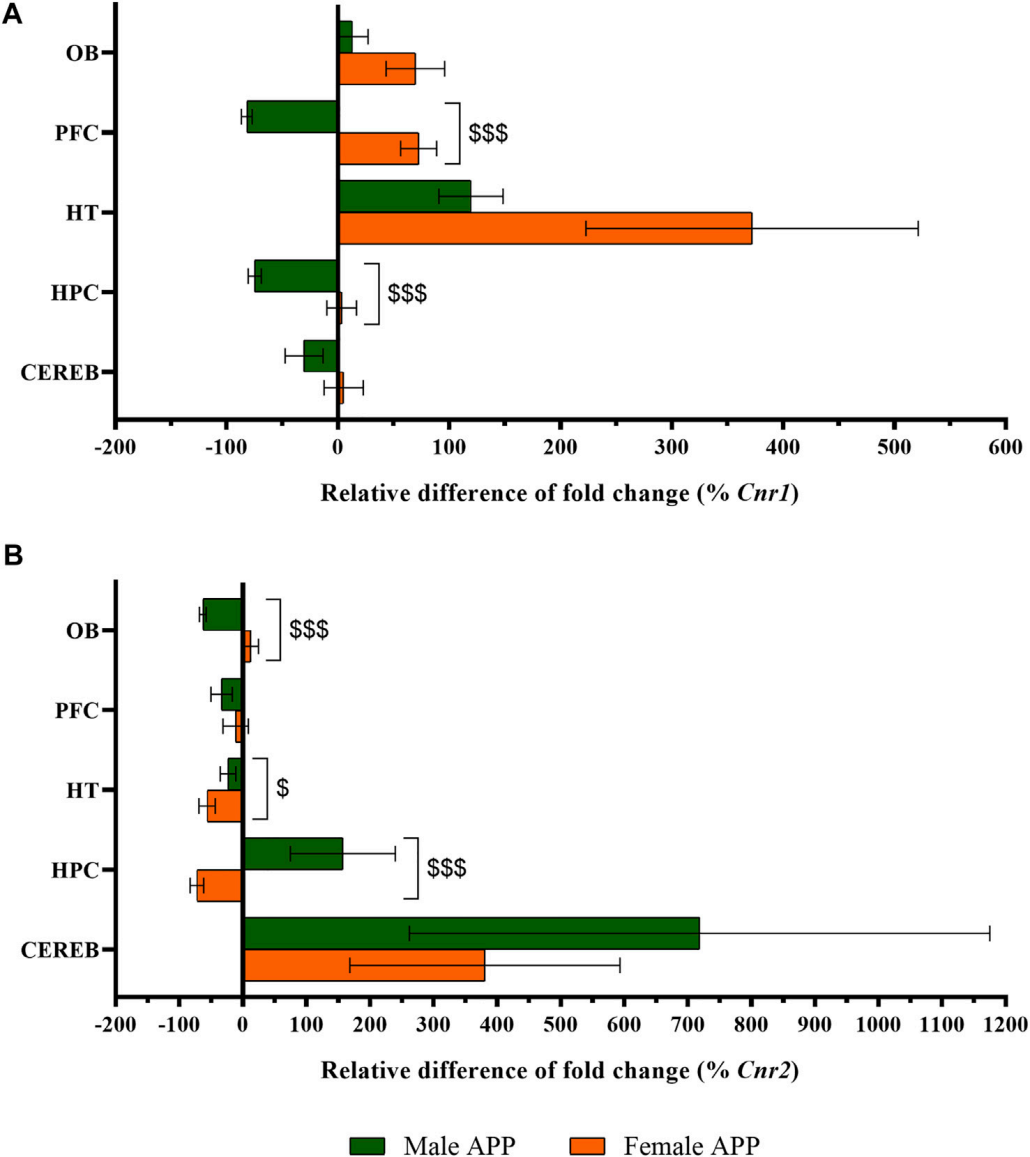
Sex-differences in CB1R and CB2R gene expression between WT and APP/PS1 mice (male *n* = 9–10, female *n* = 9–10). Expression of **(A)** *Cnr1* and **(B)** *Cnr2*. The *X* axis represents the relative difference of the fold change in percentage. The *Y* axis shows the five brain-regions analyzed: olfactory bulb (OB), prefrontal cortex (PFC), hypothalamus (HT), hippocampus (HPC), and cerebellum (CEREB). Values are expressed as mean ± SEM. $ *p* < 0.05, $$$ *p* < 0.001, vs. female APP mice (sex effect); (Mann–Whitney test).

The statistical analysis of *Cnr1* revealed that male APP/PS1 mice presented lower gene expression levels in both prefrontal cortex (U = 11.0, *p* < 0.05) and hippocampus (U = 10.0, *p* < 0.05) compared to WT mice. In contrast, higher levels of *Cnr1* were found in the hypothalamus (U = 11.0, *p* < 0.05) (Figure 4A). In females, APP/PS1 mice showed significantly greater mRNA levels of *Cnr1* in the olfactory bulb (U = 14.0, *p* < 0.05), prefrontal cortex (U = 17.0, *p* < 0.05), and hypothalamus (U = 6.0, *p* < 0.05) than WT mice (Figure 4B). Regarding *Cnr2*, male APP/PS1 mice displayed significantly higher levels in cerebellum (U = 14.0, *p* < 0.05) compared with WT mice, while no significant differences were found in the rest of the brain-regions analyzed (Figure 4C). Female APP/PS1 mice also showed a significant enhancement of *Cnr2* levels in the cerebellum (U = 17.0, *p* < 0.05) with respect to WT group, but less expression in the hippocampus (U = 20.0, *p* < 0.05) (Figure 4D). Additionally, we analyzed the impact of sex on those alterations found in both *Cnr1* and *Cnr2* gene expression levels. Mann-Whitney U test revealed a main effect of sex in the prefrontal cortex (U = 0.000, *p* < 0.001) and hippocampus (U = 0.000, *p* < 0.001) for *Cnr1* expression (Figure 5A), whereas a significant effect of sex was found in the olfactory bulb (U = 2.0, *p* < 0.001), hypothalamus (U = 13.0, *p* < 0.05) and hippocampus (U = 4.0, *p* < 0.001) for *Cnr2* expression (Figure 5B). Altogether, these data suggest that the gene expression levels of cannabinoid receptors are already altered in a sex-dependent manner during the presymptomatic period of the AD-like disease present in APP/PS1 mice.

#### 3.2.2 2-AG Signaling

**FIGURE 6:**
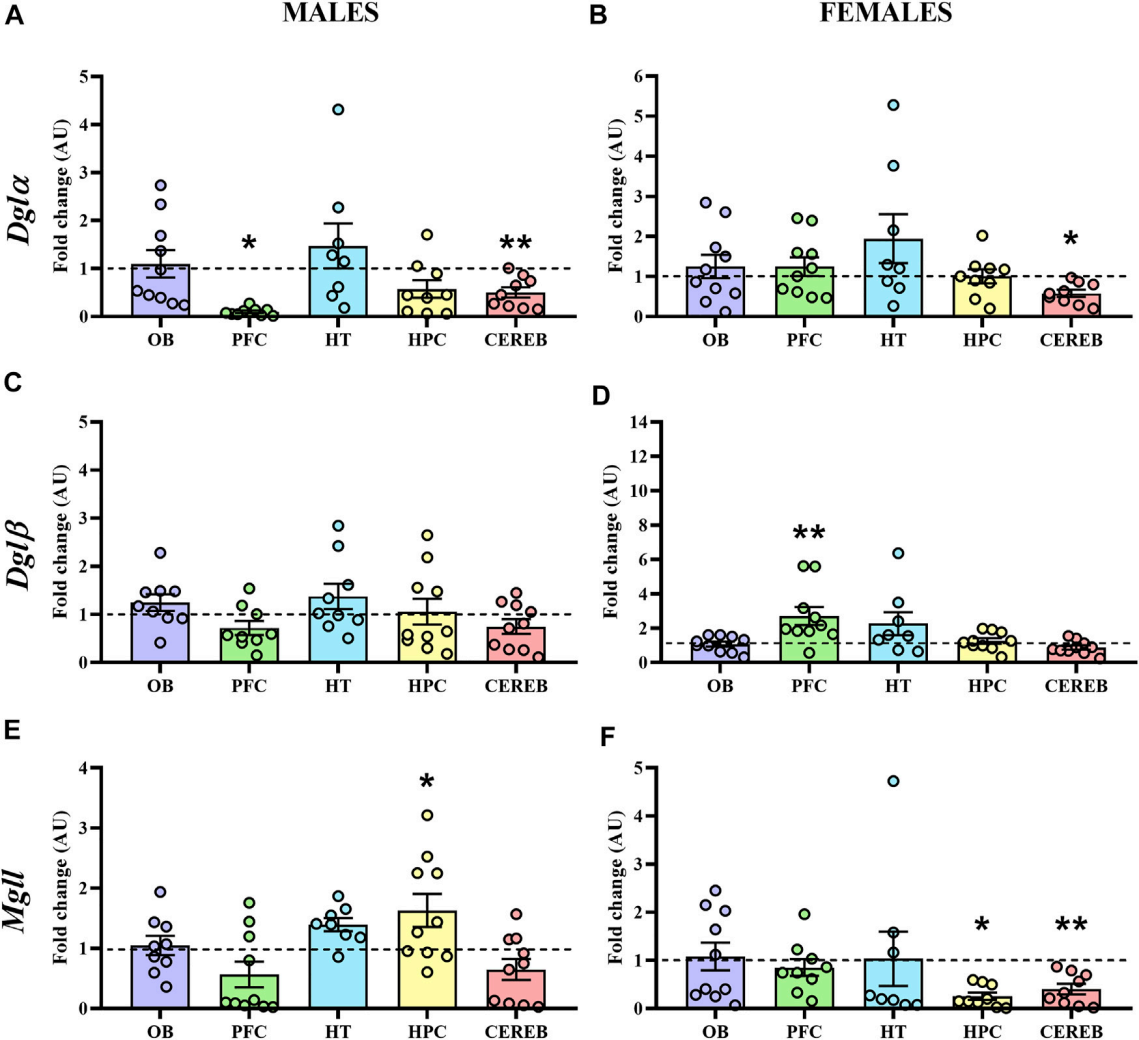
Gene expression of the enzymes involved in the 2-AG signaling in WT (male *n* = 10; female *n* = 10) and APP/PS1 (male *n* = 10; female *n* = 10) mice at 3 months of age. RT-qPCR assay was performed to analyze the expression of *Dglα*, *Dglβ* and *Mgll* in **(A,C,E)** males and **(B,D,F)** females, respectively. The *X* axis shows the five brain-regions analyzed: olfactory bulb (OB), prefrontal cortex (PFC), hypothalamus (HT), hippocampus (HPC), and cerebellum (CEREB). The *Y* axis shows the mRNA expression as a fold change comparing male and female APP/PS1 mice with its corresponding WT (represented as a dashed line). Values are presented as the mean ± SEM. *p < 0.05, **p < 0.01, vs. WT group (genotype effect); (Mann–Whitney test).

In male APP/PS1 mice*, Dglα* expression levels were significantly decreased in both prefrontal cortex (U = 17.0, *p* < 0.05) and cerebellum (U = 11.0, *p* < 0.05) with respect to controls (Figure 6A), whereas female APP/PS1 mice only presented this significant reduction in the cerebellum (U = 15.0, *p* < 0.05) (Figure 6B). As for *Dglβ*, we did not observe any significant change in male mice (Figure 6C), while female APP/PS1 mice presented significantly enhanced expression in the prefrontal cortex (U = 10.0, *p* < 0.05) in comparison with WT mice (Figure 6D). The statistical analysis of the *Mgll* expression revealed that male APP/PS1 mice presented significantly higher expression in the hippocampus (U = 16.0, *p* < 0.05) compared to controls (Figure 6E). However, female APP/PS1 mice showed a significant reduction in the hippocampus (U = 17.0, *p* < 0.05) and cerebellum (U = 11.0, *p* < 0.05) (Figure 6F). These results indicate that the three brain regions where 2-AG signaling could be most affected in APP/PS1 mice at a presymptomatic stage are the prefrontal cortex, hippocampus, and cerebellum.

#### 3.2.3 AEA Signaling

**FIGURE 7:**
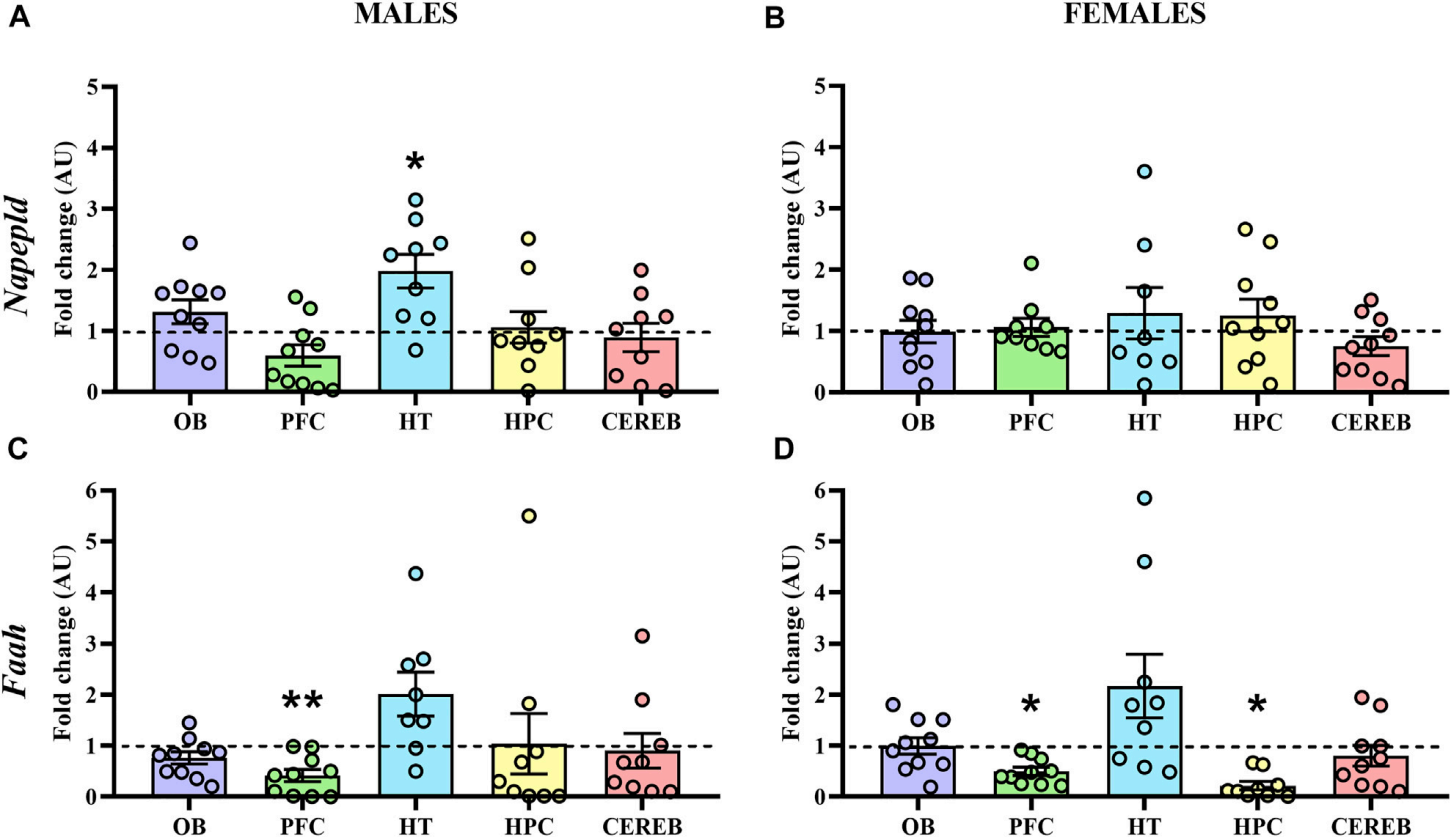
Gene expression of the AEA metabolizing enzymes in WT (male *n* = 10; female *n* = 10) and APP/PS1 (male *n* = 10; female *n* = 10) mice at 3 months of age. RT-qPCR assay was performed to analyze the expression of *Napepld* and *Faah* in **(A,C)** males and **(B,D)** females, respectively. The *X* axis shows the five brain-regions analyzed: olfactory bulb (OB), prefrontal cortex (PFC), hypothalamus (HT), hippocampus (HPC), and cerebellum (CEREB). The *Y* axis shows the mRNA expression as a fold change comparing male and female APP/PS1 mice with its corresponding WT (represented as a dashed line). Values are presented as the mean ± SEM. *p < 0.05, **p < 0.01, vs. WT group (genotype effect); (Mann–Whitney test).

Finally, we analyzed the mRNA levels of *Napepld* and *Faah*, the endocannabinoid metabolizing enzymes of AEA. Concerning *Napepld*, the statistical analysis only detected a significant increase of its expression in the hypothalamus (U = 13.0, *p* < 0.05) of male APP/PS1 mice compared to the WT (Figure 7A), with no significant changes in females (Figure 7B). On the other hand, we found a significant decrease in the gene expression levels of *Faah* in the prefrontal cortex (U = 16.0, *p* < 0.05) of male APP/PS1 mice compared to the WT (Figure 7C). In female APP/PS1 mice, a decrease of *Faah* expression in the prefrontal cortex (U = 15.0, *p* < 0.05) and hippocampus (U = 15.0, *p* < 0.05) was significantly found in relation to its WT littermates (Figure 7D). These results point out that the prefrontal cortex, and to a lesser extent the hypothalamus, are the brain regions where most potential differences are found regarding AEA signaling.

## 4 Discussion

To develop new therapeutic strategies against complex brain diseases such as AD, there is an urgent need to better understand their pathophysiology considering potential factors that have been unexplored such as differences between sexes or brain regions. This study, by combining behavioral and gene expression analysis in APP/PS1 mice, aimed to characterize the ECS in male and female WT and APP/PS1 mice during a presymptomatic period (3 months of age) considering the sex variable in the brain regions analyzed. Strikingly, we found interesting differences in the expression levels of several components of the ECS in different brain regions and between sex, which could indicate that the ECS is already altered in APP/PS1 mice at presymptomatic stages becoming an early event contributing to AD pathology or a potential predictive biomarker.

Transgenic AD mouse models are extensively used because they partially mimic the behavioral and molecular dysfunctions found in AD patients. The pathological onset of AD in humans and animal models is generally silent as it takes some time from the first disease-related alteration to the declining of behavioral skills (McKhann et al., 2011). Thus, a better understanding of this presymptomatic period could bring some new therapeutic approaches to slow down disease progression (DeKosky et al., 2008; Holmes et al., 2008; Martin et al., 2008). Indeed, current therapeutic failures might be the result of an intervention that is either too late (i.e., with established symptoms) or that targets processes that are not directly involved in the onset of the disease or progression (Hyman, 2011). However, initiating preventive treatments in presymptomatic periods requires a better comprehension of what distinguishes preclinical AD from normal aging, and which potential alterations are causally associated with the pathophysiology of the disease. Previous studies suggested that the ECS might be a therapeutic target against AD, but it is still unknown whether it is an altered system because of the disease or it is indeed a possible cause of AD pathophysiology. Our gene expression results give some insights on the alterations of the ECS during the presymptomatic period in APP/PS1 mice. These results are quite intriguing as most of them are found in brain areas that are evidently affected in AD such as the hippocampus or prefrontal cortex. Even so, whether changes in mRNA expression have any biological meaning is still a matter of debate (Liu et al., 2016). Therefore, although differences in mRNA(s) of different ECS components can indicate an early alteration of this system in AD, future studies will need to assess protein expression and/or associated metabolites to fully establish the ECS relevance during the AD presymptomatic period. Moreover, it would be also interesting to analyze the specific localization of ECS components in different stages of AD pathology. For instance, it has been already shown an altered membrane localization of CB1R in AD pathology, which has an impact on the coupling to its effector proteins and in its inhibitory control (Maccarrone et al., 2018).

A potential sexual dimorphism has been identified in AD (Aso et al., 2012, 2016, 2018; Haghani et al., 2012; Jiao et al., 2016). For example, female mice exhibit higher pathological levels of Aβ40 and Aβ42 peptides and show differences in the nature of their behavioral impairment (Bilkei-Gorzo, 2014; Cheng et al., 2014; Coles et al., 2020). Importantly, analyzing the link between AD and the ECS considering the sex variable is especially interesting as there are sex differences in humans regarding the availability and sensitivity of the ECS (Laurikainen et al., 2019; Wagner, 2016), which could be also present in rodents (Rubino and Parolaro, 2011; Calakos et al., 2017). Therefore, given the relevance of sex in AD, sex-dependent analyses minimize the loss of information that can occur when sex is not included as a variable in previous studies. Notably, our results indicate a sex effect regarding the mRNA levels of cannabinoid receptors that need to be further explored in the future. For example, these sex-dependent differences in CB1R and CB2R gene expression are mostly found in brain regions involved in learning and memory processes, which could be one of the reasons explaining the potential behavioral differences at the later stages between males and females. However, a better characterization of the cognitive skills of APP/PS1 mice at later stages is required in the future to fully link these sex-dependent changes on mRNA expression and the potential behavioral differences.

Our results showed that CB1R mRNA levels are decreased in both hippocampus and prefrontal cortex of APP/PS1 male mice. Accordingly, alterations in CB1R expression have been reported in both human AD brains (Manuel et al., 2014; Talarico et al., 2018; Berry et al., 2020; Medina-Vera et al., 2020) and AD mouse models (Kalifa et al., 2011; Aso et al., 2012; Bedse et al., 2014a). In contrast, an increase of CB1R immunostaining in the neocortex of APP/PS1 male mice at 3 months (Aso et al., 2012) or unchanged CB1R protein expression in the hippocampus and prefrontal cortex at 2 and 4 months of age in other AD animal models (Bedse et al., 2014b; Maccarrone et al., 2018) have been recently described. Discrepancies between these different studies might be due to the different techniques used, the AD mouse model, the age studied, or the brain region analyzed. In addition, although some studies showed no changes in the expression levels, the specific CB1R localization and signaling could be different between genotypes impacting AD pathology (Maccarrone et al., 2018). Nevertheless, our results indicate sex differences that have not been fully studied in previous studies. In addition, the reduction of CB1R gene expression in cognitive-related brain regions in male APP/PS1 mice at 3 months of age could participate in the cognitive deficits observed in later stages, and the sex differences found could point to distinct alterations in the AD progression of male and female APP/PS1 mice.

CB2R alterations have also been detected in both AD patients’ brains (Benito et al., 2003; Solas et al., 2013; Talarico et al., 2018; Berry et al., 2020; Medina-Vera et al., 2020) and AD mouse models (Medina-Vera et al., 2020). The present study revealed a CB2R reduction in the hippocampus of female APP/PS1 mice, while male APP/PS1 mice tended to have higher mRNA levels. Previous studies indicate that CB2R levels are increased specifically in the microglia surrounding Aβ plaques and astrocytes as a result of inflammatory damage (Benito et al., 2003; Ramírez et al., 2005; Solas et al., 2013; Aso et al., 2016; Medina-Vera et al., 2020), although the distinction between males and females was missing. Considering these facts, an interesting hypothesis to be tested in the future is whether CB2R expression levels could differ between male and female APP/PS1 mice because of a sex-dependent level of neuroinflammation. Our current results show no differences regarding astrogliosis or microgliosis at 3 months of age although the link between CB2R and chronic neuroinflammation has to be further explored both at early and late stages of the disease. In this sense, it has been previously demonstrated that CB2R activation reduces inflammatory processes, facilitates Aβ removal, modulates tau hyperphosphorylation and oxidative stress, and ameliorates cognitive abilities in AD mouse models (Ramírez et al., 2005; Van Der Stelt et al., 2006; Esposito et al., 2007; Fakhfouri et al., 2012; Aso et al., 2013; Wu et al., 2013; Aso et al., 2016; Li et al., 2019). Even though, whether this neuroinflammation could be linked with the differences in cognitive deficits is unknown. Altogether, our results regarding CB1R and CB2R mRNA levels indicate that both cannabinoid receptors could be useful biomarkers or therapeutic targets during the presymptomatic stage of AD.

Alterations of the enzymes involved in the biosynthesis and degradation of endocannabinoids (eCBs) have also been reported in AD patients (Benito et al., 2003; Mulder et al., 2011; Bedse et al., 2014a; Berry et al., 2020) and AD mouse models (Piro et al., 2012; Bedse et al., 2014b; Medina-Vera et al., 2020). In our study, we also analyzed the endocannabinoid anabolic and catabolic enzymes finding the 2-AG signaling altered in the prefrontal cortex and cerebellum, and the AEA signaling altered in the prefrontal cortex and hypothalamus of APP/PS1 mice. These alterations could be linked with alterations in the levels of the eCBs, specifically 2-AG and AEA. Indeed, the possible overproduction of eCBs has already been described in other studies, which could probably reflect an anti-inflammatory response due to neuronal damage (Marsicano et al., 2003; Chen et al., 2012; Medina-Vera et al., 2020). Notably, it has been demonstrated that the inhibition of MAGL or FAAH, which increases the levels of eCBs indirectly, suppresses neuroinflammation and prevents neurodegeneration against harmful insults (Chen et al., 2012; Piro et al., 2012; Ren et al., 2020). Indeed, FAAH deletion in microglial cells decreased inflammatory processes (Tanaka et al., 2019) and specific FAAH inhibitors are able to drive these cells towards an anti-inflammatory phenotype in the context of AD (Grieco et al., 2021). This is relevant as decreased FAAH gene expression levels have been found in the prefrontal cortex and hippocampus of female mice. On the other hand, we speculated that male APP/PS1 at 3 months of age present a 2-AG deficiency in the prefrontal cortex in contrast to female APP/PS1, which likely have higher 2-AG levels in the hippocampus and prefrontal cortex. However, all these hypotheses should be tested in future projects to better understand the impact of eCBs in AD pathophysiology.

Although the main AD symptoms are associated with cognitive decline, AD is also accompanied by disturbances in energy metabolism (Vercruysse et al., 2018; Zheng et al., 2018), olfactory alterations (Murphy, 2019; Mitrano et al., 2021), and motor coordination deficits (Wagner et al., 2019). Interestingly, the ECS has been involved in all these functions (Busquets-Garcia et al., 2015; Roger, 2015). Thus, we decided to analyze the ECS components in brain regions that have not been extensively studied in the context of AD such as the hypothalamus, the olfactory bulb, and the cerebellum, which are involved in all these functions. Although further research is needed to elucidate the possible link between AD, the ECS, and the metabolic, olfactory, or motor alterations, our results pointed out that alterations of the ECS components in the hypothalamus, olfactory cortex and cerebellum could appear in the early stages of disease progression in the APP/PS1 mice and could contribute to some of the behavioral alterations that are associated with AD (Zheng et al., 2018; Murphy, 2019; Wagner et al., 2019) but have been less studied.

We must acknowledge some open questions arising from our study. Firstly, the presymptomatic stage has been defined as the period without major behavioral alterations and the lack of astrogliosis and microgliosis, which are hallmarks of AD pathology at later stages. Although previous studies show the absence of amyloid plaques at this stage (Garcia-Alloza et al., 2006; Aso et al., 2012), future analysis must extend these evaluations by exploring other neuropathological events such as levels of soluble amyloid peptides or alterations on synaptic loss at these early stages. Secondly, the impact of sex hormones on the sex differences observed was not directly assessed in the present study. The role of sex hormones on neuroscience research in general (Shansky, 2019), or in the cannabinoid field (Fattore and Fratta, 2010) in particular, has been widely discussed. Future experiments must address whether testosterone and/or estrogens are responsible or contribute to mRNA expression levels differences when comparing male and female APP/PS1 mice. In the third place, as discussed above, although clear and interesting findings arise from the present work, future experiments will determine if differences in mRNA levels are accompanied by changes at the protein level and from a functional point of view. In the last place, future experimental designs must aim to causally link these early alterations in the ECS with the later behavioral alterations with especial emphasis on sex-dependent behavioral phenotypes. Although a mild deficit was observed in the spontaneous alternation in male APP/PS1 mice, we considered that we are still in the early stages of AD progression and a behavioral assessment at the later stages is required as all the other behavioral tests show no genotypic differences. Furthermore, with the present results, is difficult to attribute sex-dependent differences in this task as control female mice do not show proper behavior in the Y maze and figuring out the reason is out of the scope of the present project. Although we should consider these aspects in future works, the present study revealed that the main components of the ECS are already altered in APP/PS1 mice at the presymptomatic stage in a sex-dependent manner, suggesting that it could be an early event contributing to AD pathology, or a potential predictive biomarker. Future similar analysis in presymptomatic patients or other mouse models must confirm the early alterations of the ECS in AD. In addition, future experiments can also target the analysis to other interesting receptors or enzymes that have been linked with ECS components forming the so-called “endocannabinoidome.”

## Data Availability

The original contributions presented in the study are included in the article/Supplementary Material, further inquiries can be directed to the corresponding author.
